# 3D Bayesian cluster analysis of super-resolution data reveals LAT recruitment to the T cell synapse

**DOI:** 10.1038/s41598-017-04450-w

**Published:** 2017-06-22

**Authors:** Juliette Griffié, Leigh Shlomovich, David J. Williamson, Michael Shannon, Jesse Aaron, Satya Khuon, Garth L. Burn, Lies Boelen, Ruby Peters, Andrew P. Cope, Edward A. K. Cohen, Patrick Rubin-Delanchy, Dylan M. Owen

**Affiliations:** 1 Department of Physics and Randall Division of Cell and Molecular Biophysics, King’s College London, London, UK; 2 Department of Mathematics, Imperial College London, London, UK; 3 Advanced Imaging Center, Howard Hughes Medical Institute Janelia Research Campus, Ashburn, Virginia USA; 4 Department of Medicine, Imperial College London, London, UK; 5 Department of Immunology, Infection and Inflammatory Disease, King’s College London, London, UK; 6 Department of Statistics, University of Oxford, Oxford, UK

## Abstract

Single-molecule localisation microscopy (SMLM) allows the localisation of fluorophores with a precision of 10–30 nm, revealing the cell’s nanoscale architecture at the molecular level. Recently, SMLM has been extended to 3D, providing a unique insight into cellular machinery. Although cluster analysis techniques have been developed for 2D SMLM data sets, few have been applied to 3D. This lack of quantification tools can be explained by the relative novelty of imaging techniques such as interferometric photo-activated localisation microscopy (iPALM). Also, existing methods that could be extended to 3D SMLM are usually subject to user defined analysis parameters, which remains a major drawback. Here, we present a new open source cluster analysis method for 3D SMLM data, free of user definable parameters, relying on a model-based Bayesian approach which takes full account of the individual localisation precisions in all three dimensions. The accuracy and reliability of the method is validated using simulated data sets. This tool is then deployed on novel experimental data as a proof of concept, illustrating the recruitment of LAT to the T-cell immunological synapse in data acquired by iPALM providing ~10 nm isotropic resolution.

## Introduction

Images from conventional fluorescence microscopy techniques consist of pixelated intensity distributions representing the local density of fluorophores over the sample. These techniques are limited by the diffraction of light to a resolution of about 200 nm. Although coarse information can be extracted from such images, they are unsuitable for the study of nanoscale molecular organisation. To circumvent the diffraction limit, a new family of super-resolution fluorescence microscopy techniques has emerged. Although these encompass a variety of methods based on different strategies, they all achieve a resolution beyond the diffraction limit of around 200 nm. The approach of single molecule localization microscopy (SMLM), such as photo-activated localisation microscopy (PALM)^1, 2^ and stochastic optical reconstruction microscopy (STORM)^3, 4^, achieve enhanced resolution by temporally separating the emission of individual fluorophores. As only a sparse subset of fluorophores are stochastically turned on (i.e. visible) in each frame, their associated point spread functions (PSFs) do not overlap on the detector, allowing the precise localisation of each emitter as the centre of their PSF^5^. To reconstruct the full fluorophore population distribution, fluorophores are turned on and off and acquired in successive frames over time, with each emitter’s localised coordinates recorded in each frame. The reconstructed image data therefore consists of a list of localisation coordinates from all data frames, as well as the associated localisation uncertainties. As the pointillist nature of SMLM datasets differs so markedly from pixelated intensity images produced in conventional microscopy, the analysis of SMLM images requires the development of new analysis tools.

SMLM techniques usually rely on the optical sectioning of the sample as a way to reduce background which otherwise would be detrimental to the precise localisation of fluorescence events. The resulting SMLM data set consists of the 2D projection of the illuminated volume in the sample (typically ~100 nm depth in the case of total internal reflection fluorescence (TIRF) illumination). The penalty for the improved signal to noise ratio through such approaches is that any 3D information from the sample is lost, limiting the interpretation of the data. 3D SMLM offers a unique insight into the organisation of molecules proximal to the coverslip. A number of techniques have now emerged to achieve 3D SMLM acquisition, including the use of astigmatic lenses^6, 7^, biplane imaging^8^, helical point spread functions^9^ and interferometric approaches such as iPALM^10^. Typically, a highly-inclined and laminated optical sheet (HILO) is used for the illumination, which allows imaging up to 600 nm into the sample^11^. iPALM, by using two objectives and the interference properties of light, is essentially unique in achieving a localisation precision in the z dimension comparable to that in x and y, i.e. 10 to 30 nm depending on the fluorophore used^12^. The resulting data sets are lists of coordinates (*x*, *y*, *z*) and their theoretically estimated uncertainties (*σ*_*x*_, *σ*_*y*_, *σ*_*z*_), which the cluster analysis method presented here takes as input. Here, the uncertainties are calculated according to the method of Thompson *et al*.^13^, as this is the most widely applied method in the literature, however, other methods are equally applicable to our analysis.

SMLM imaging has highlighted the importance of molecular clustering in the regulation of cellular processes. Cell signalling, for example, immune cell migration and activation, rely on protein clustering and translocation^14–16^. Key signalling pathways have been shown to be triggered by such spatio-temporal reorganisation of molecules^17, 18^. Hence, in parallel with the development of SMLM, analysis tools have emerged to quantify this clustering behaviour. Although many analysis strategies have been adapted for 2D SMLM (e.g. pair correlation^19, 20^, Ripley’s K-function^21, 22^ and Voronoi tessellation^23^), 3D analytical tools remain limited. Here, we present a new 3D cluster analysis method, based on a combination of the localised Ripley’s K-function^24^, topographic prominence (TP)^25^, and a Bayesian statistical model^26, 27^. We show that this method is efficient, robust and accurate over a range of simulated data sets. The added value of such quantification is demonstrated using novel experimental data concerning T cell signalling, specifically, showing recruitment of LAT vesicles to the T cell immunological synapse.

T cell activation through the T cell receptor (TCR), typically initiated though interactions with antigen-presenting cells (APCs), has been shown to result in modification of protein clustering at the cell-cell interface (the immunological synapse)^28, 29^ and is vital for mounting an effective immune response. Linker for Activation of T cells (LAT) is an integral transmembrane protein which serves as an early signalling scaffold^30, 31^. It has been shown to be pre-clustered in resting T cells, with clustering increasing following activation^32–34^. Here, we explore LAT clustering at the T cell immunological synapse in 3D using iPALM and quantitative Bayesian cluster analysis. We show that the increase in LAT clustering observed in 2D results, at least in part, from the recruitment of LAT vesicles to the immunological synapse from a deep intracellular pool, distinct from the membrane population^35^. More generally, with 3D SMLM becoming a regularly used tool to address biological questions, the development of an accurate and robust 3D cluster analysis method, as presented here, is an important and necessary advance.

## Results

The cluster analysis method presented here provides detailed descriptors on clustering for 3D pointillist datasets such as those generated by PALM or STORM. While it has been developed in the context of 3D SMLM, theoretically any pointillist data set (i.e. *x*, *y*, *z*, *σ*_*x*_, *σ*_*y*_, *σ*_*z*_), can be given as input. Our cluster analysis tool consists of a model-based Bayesian statistical method^26^. The use of a model provides a method free of arbitrary user-selected analysis parameters, replacing them with well-defined Bayesian priors. Our method generates a number of cluster proposals for each region of interest, which are scored against the Bayesian model. It takes full account of the localisation uncertainty (*σ*_*x*_, *σ*_*y*_, *σ*_*z*_) of each point in its attribution (or not) to a cluster. The proposal mechanism is based on a localised version of Ripley’s K-function and a topographic prominence thresholding approach shown to be advantageous for cluster analysis of SMLM^25^. The highest-scoring cluster proposal is then identified, from which key cluster descriptors are extracted.

The generative model considers a 3D region of interest (ROI) containing clustered and non-clustered localisations. The probability that a localisation is non-clustered is set, by the user, as a prior parameter in the model, by default 50%. In what follows, we distinguish the true molecular position, which is not observed, from the observed, noisy, localisation. The molecular positions within a cluster are assumed to follow a spherical Gaussian distribution, whose standard deviation (which we call the “radius” of the cluster) is drawn from a user-specified histogram of cluster sizes. The model reflects the *a priori* knowledge on the molecular distribution. This approach is very different to setting user-defined parameters as required for conventional cluster analysis by methods such as localised Ripley’s K-function, tessellation or DBSCAN. The model consists of a probability distribution, with well-defined statistical interpretation.

Previous studies on protein clustering with SMLM, indeed suggest that circular Gaussian clusters, for the most part, recapitulate accurately the underlying distribution^27^. Moreover, to an extent, most structures can be approximated by a circular Gaussian or sum of circular Gaussians. However the model is not suited to data sets that are not recapitulated at all by the existing model (for instance fibres, such as actin filaments), in which case the analysis would lead to the detection of clusters under estimating the size of the underlying structures.

The cluster centres, as well as the non-clustered molecular positions, are assumed to follow a completely spatially random (CSR) distribution in x and y. To account for the specificities of 3D SMLM, we introduce a non-CSR distribution for the localisations in the z dimension. For example, depending on the biological phenomenon studied, molecular positions may be heavily biased towards being proximal to the coverslip. A Beta distribution is fit to the overall density of localisations in the z dimension for each ROI, which is then used as the prior distribution for the cluster centres and non-clustered molecular positions in the z dimension. This step ensures the generation of a data specific model, likely to mimic more accurately experimental data sets and hence lead to a more reliable estimation of the clustering. Finally, under the generative model, every molecular position is then subjected to a Gaussian perturbation (mimicking the localisation process), whose standard deviation in each dimension is taken from the provided localisation precision triple.

A large number of possible cluster proposals are generated, and scored according to this model. The cluster proposal routine itself consists of two major stages. First, a 3D density estimate based on Ripley’s K-function^21, 24^ is attributed to each localisation within the analysed ROI, which is treated as an indicator of local clustering. Here, a linearised and localised version of Ripley’s K-function is used. For each localisation *j* = *1*, …, *n*, this is defined as:

$${L}_{3D}{(r)}_{j}=\sqrt[3]{\frac{3V}{4(n-1)\pi }\sum _{i=1}^{n}{\delta }_{ij}}$$

where *V* is the volume of the ROI, and where *δ*_*ij*_ is 1 if *i* ≠ *j* and the distance between points *i* and *j* is less than *r*, and zero otherwise. For each localisation, *j*, therefore, the number of localisations encircled within a sphere of radius *r*, centred on *j*, is calculated. This is then normalised by the total localisation density in the ROI (the total number of localisations within the ROI, *n*, divided by the ROI volume, *V*). The value is then linearised by taking the cube root. Edge effects are corrected using a 3D toroidal wrap. Only localisations with L_3D_(*r*) above the expected value for a CSR distribution plus one standard deviation are considered as potentially part of a cluster. The second step consists of identifying local maxima within the pointillist array of L_3D_(*r*) values and applying a TP based threshold. TP, previously demonstrated to increase accuracy in cluster identification was redefined here in the context of pointillist data sets. Each maximum above this threshold is associated with a cluster (See Supplementary Information).

Thus, a single value of *r* (defining the L_3D_(*r*) value) and TP threshold *T* results in a cluster proposal for an ROI. By varying *r* and *T* over a user-specified range, many thousands of proposals are generated and scored against the model. The highest scoring proposal is the one with the maximum *a posteriori* probability of the data given the model. The highest scoring proposal is retained and key cluster descriptors extracted, such as the positions (*x*, *y*, *z*) of all of the detected clusters, the cluster radii, the percentage of localisations in clusters, the number of clusters and the number of localisations per cluster.

**Figure 1:**
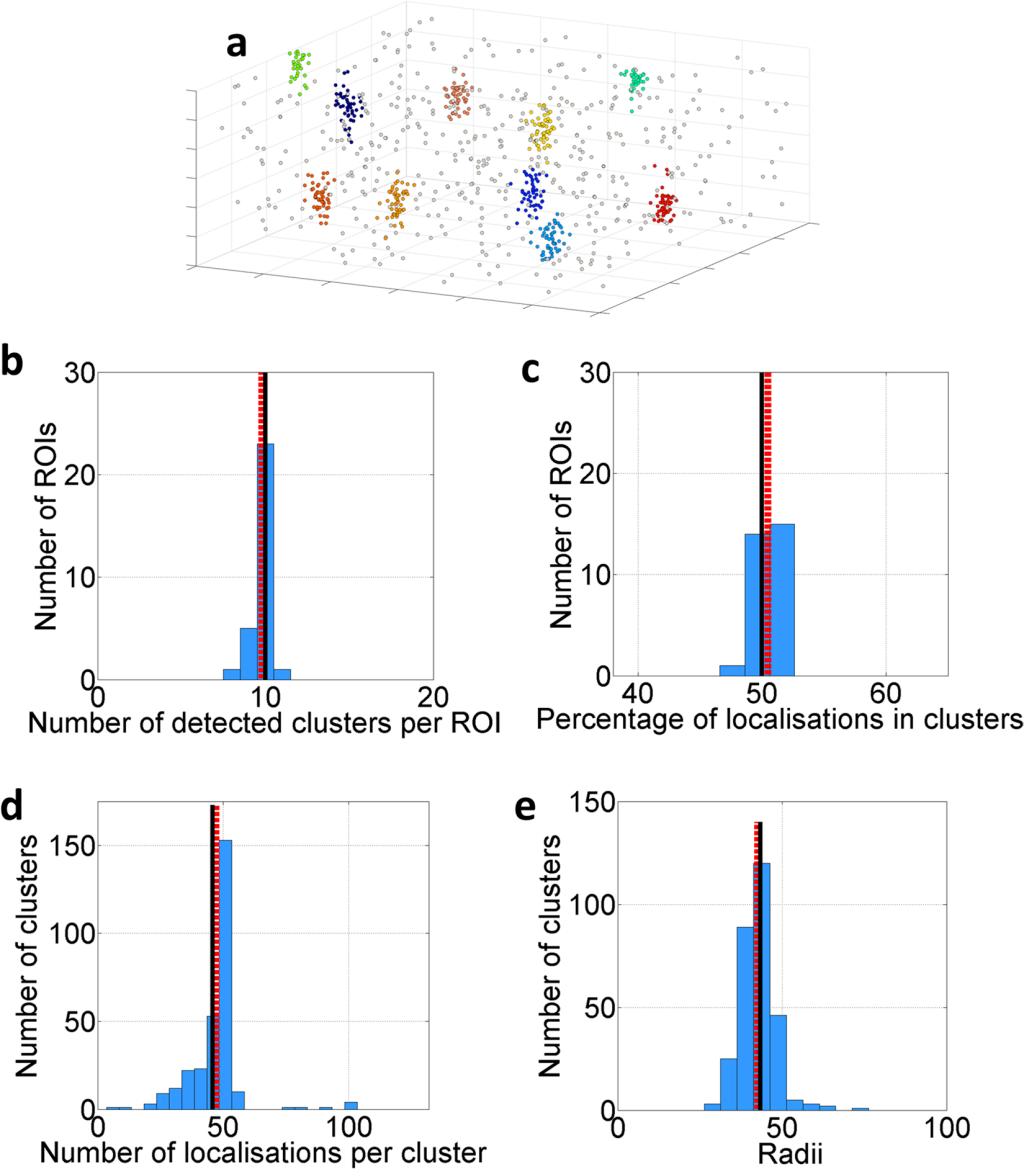
3D Bayesian cluster analysis of n = 30 simulated data sets in the Standard Condition. (**a**) Representative 3D cluster map with the simulated clusters depicted in colour and non-clustered localisations in grey. (**b**) Number of detected clusters per ROI, (**c**) percentage of localisations detected in clusters, (**d**) number of detected localisations per cluster and (**e**) cluster radii﻿ (nm)﻿. In each case (**b–e**), the simulated values (black solid line) and mean detected value (red dashed line) are presented.

We demonstrate the performance of our method on simulated data. We define the Standard Condition as a 3000 nm × 3000 nm × 600 nm volume (*x*, *y*, *z*) containing 10 clusters, each with a standard deviation (radius) of 30 nm placed with uniform probability throughout the volume. There are 50 molecules per cluster. Within this volume, an equal sized population of non-clustered molecules are overlaid such that there are 1000 molecules in total and 50% of molecules are in clusters. The distribution of molecules in z is uniform. Each molecular coordinate is then scrambled by independent Gaussian measurement error, with standard deviation drawn from a Gamma distribution with mean 30 nm to mimic the localisation process. The Standard Condition aims to replicate typical experimental data sets, and a representative analysed cluster map is shown in Fig. 1a. The performance of the algorithm is evaluated by four cluster descriptors, number of clusters per map (Fig. 1b), percentage of localisations in clusters (Fig. 1c), number of localisations per clusters (Fig. 1d) and cluster radii (Fig. 1e), from 30 simulated ROIs. In all four cases, the estimated cluster descriptors agree with the true simulated values.

In many SMLM data sets, the protein of interest is directly or indirectly membrane associated leading to a non-uniform distribution of localisations in z. A 3D clustering algorithm for SMLM must be able to cope with this phenomenon, and our algorithm achieves this by modelling the distribution in z explicitly. We demonstrate this on simulated data in which the z distribution of the non-clustered localisations follows a Beta distribution with parameters α = 2, β = 5, showing highly robust results (SI Appendix Fig. S1). Other existing techniques such as DBSCAN or tessellation, because they do not rely on a model, are unlikely to provide reliable descriptors in the case of an uneven background as described by Rubin-Delanchy *et al*. in the case of 2D SMLM data^26^. This characteristic of the Bayesian approach is key to quantifying experimental datasets as the distribution in z varies from one condition to the other. Our method is therefore appropriate for analysing the clustering of cytosolic proteins — with a homogenous distribution in z — as well as those associated with the plasma membrane. We also show that in the case of a plasma membrane proximal distribution (as simulated by using a Beta distribution with parameters 2, 20 for the distribution of localisations in z), the use of a Beta distribution fit and prior in the model enables a decrease of 16% in the number of detected artificial clusters and provides reliable descriptors (SI Appendix Fig. S2).

In biological samples, molecule-molecule interactions might not necessarily lead to a Gaussian profile spherical clusters. To verify the validity of our cluster analysis to non-circular or non-Gaussian profile clusters, we have simulated and analysed hard edge clusters as well as ellipses (ratio (1, 1, 2), randomly oriented along the x, y or z axis) in the Standard Condition. The result demonstrates the validity of our model even in the context of 3D non-Gaussian profile as well as non-circular clusters (SI Appendix Figs S3 and S4), agreeing with the 2D case^27^. We further verified the sensitivity of the analysis tool to user defined parameters by analysing the Standard Condition with the prior on the ratio of un-clustered localisations set at 25% (SI Appendix Fig. S5), and 75% (SI Appendix Fig. S6). In both cases, the analysis provides reliable descriptors that recapitulate the results obtained with the default settings, proving the robustness of model-based analysis. Finally, to measure the rate of false detection, we analysed CSR distributions, with the same total number of localisations per ROI as the Standard Condition (SI Appendix Fig. S7). Overall, only 3.1% of the localisations were attributed to clusters.

**Figure 2:**
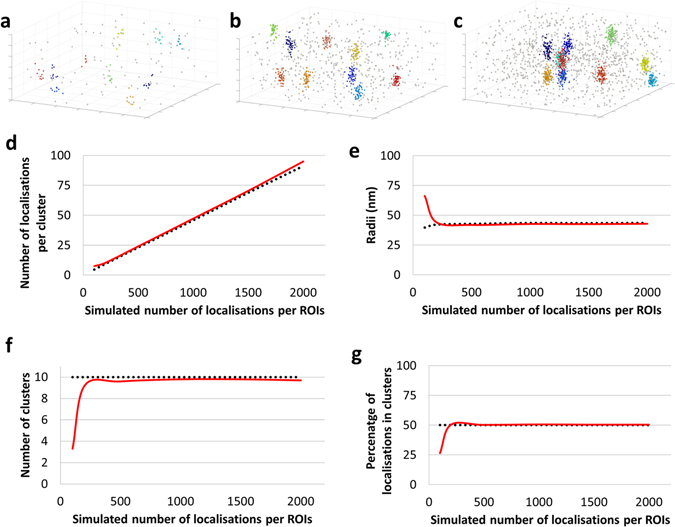
Examining the effect of increasing total number of localisations in the ROI on the Bayesian cluster analysis of simulated data sets (n = 30 simulations) from 100 localisations per ROIs to 2000 localisations per ROIs, under the Standard Conditions. Representative cluster maps with (**a**) 200, (**b**) 1000 and (**c**) 2000 localisations within the 3000 × 3000 × 600 nm ROI. (**d**) Mean number of detected localisations per cluster as a function of the number of localisations per cluster, compared to the simulated (true) value, (**e**) mean cluster radii as a function of the number of localisations per cluster, compared to the simulated (true) value, (**f**) mean number of detected clusters per ROI as a function of the number of localisations per cluster, compared to the simulated value and (**g**) mean percentage of localisations detected in clusters as a function of the number of localisations per cluster, compared to the simulated value. Red = results of the analysis, black = simulated values.

We next verify the robustness of the algorithm to different simulated cluster conditions, keeping the default priors. Starting from the Standard Condition, we vary two parameters independently; first, the total number of localisations per 3000 nm × 3000 nm × 600 nm ROI from 100 localisations to 2000 localisations (Fig. 2) and second, the percentage of non-clustered localisations from 10% to 90% (SI Appendix Fig. S8), keeping all other parameters constant. Representative example maps (from n = 30 simulations) with 200 localisations (Fig. 2a), 1000 localisations (Fig. 2b) and 2000 localisations (Fig. 2c) are shown. The analysis demonstrates that the number of localisations per cluster (Fig. 2d), cluster radii (Fig. 2e), number of clusters per ROI (Fig. 2f) and percentage of localisations in clusters (Fig. 2g) are estimated equally accurately across a range of values for the total number of localisations. The results on simulation suggest that our cluster analysis algorithm provides reliable quantification down to under 10 localisations per cluster in the case where 50% of localsiations are found in clusters. Under this limit, fewer clusters are detected, and the average number of localisations per cluster remains around 7. At very low density (i.e ~20 localisations per μm^3^), the analysis is more likely to detect clusters which overlap, explaining the increase in the average cluster radius. Similar results while varying the percentage of non-clustered localisations are displayed in SI Appendix Fig. S8, again showing that the results are largely robust to this variation.

After validating the 3D Bayesian cluster analysis method on simulated data, we tested the algorithm with experimental data. Our experimental data set consists of super-resolution microscopy images of the LAT scaffold protein in non-activated T cells and at the T cell immunological synapse at different time-points, acquired by iPALM^10^. The use of iPALM allowed imaging up to 600 nm deep into the sample above the coverslip, with an approximately isotropic uncertainty on each localisation along x, y and z. Nevertheless, the localisation precisions for each localisation were independently calculated theoretically for each dimension, and treated accordingly in the analysis. Although LAT has been investigated with conventional microscopy techniques as well as 2D SMLM before, here we present new spatio-temporal information obtained by 3D SMLM iPALM.

Conventional microscopy studies of LAT, necessarily limited in resolution, can only produce an overall and limited description of the clustering behaviour. 2D SMLM provides a clearer description of the nanoscale organisation of LAT at the IS, however, it still only produces an incomplete description of the cellular distribution. Firstly, only LAT in the direct vicinity of the membrane are detected in the case of 2D SMLM (~100 nm illuminated with TIRF), thus the analysis is lacking a description of a potential vesicular population higher up in the sample. This remains a clear limitation when trying to understand the LAT recruitment process. Also, 2D SMLM consists of a projection of ~100 nm depth information onto a 2D plane. This could in turn lead to a misinterpretation of the LAT clustering following T cell activation via the TCR-CD3 complex. Interestingly, the switch to 3D SMLM acquisition and quantification provides a unique insight into LAT reorganisation and recruitment to the IS following cell activation.

**Figure 3:**
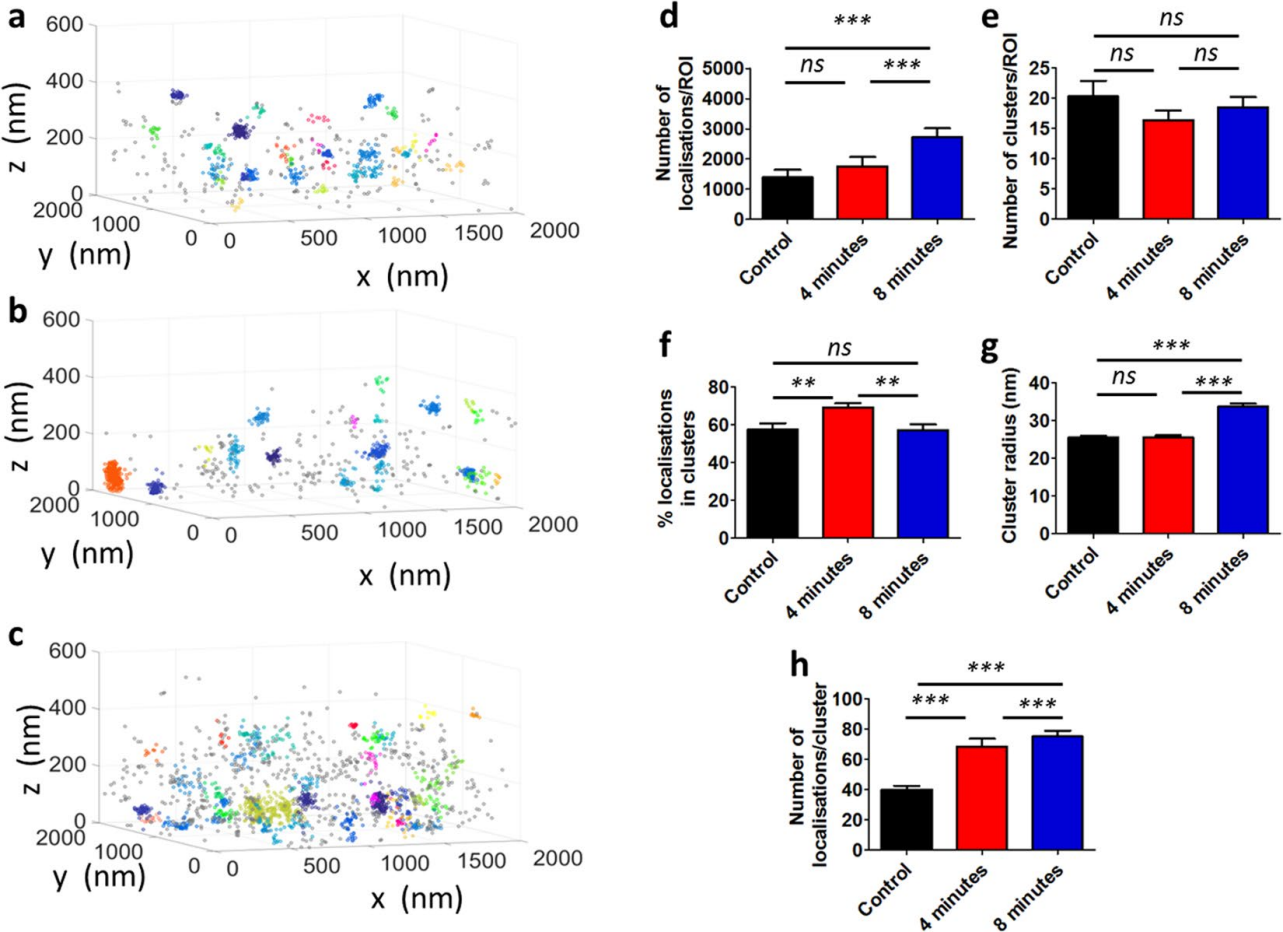
3D Bayesian cluster analysis of iPALM data of the distribution of LAT at the T cell immunological synapse. Representative cluster maps of LAT-mEos3.2 (**a**) in non-activated T cells, (**b**) in T cell synapses fixed after 4 mins, (**c**) in T cell synapses fixed after 8 mins. For each condition, (**d**) total number of localisations per ROI, (**e**) number of detected clusters per ROI, (**f**) percentage of localisations in clusters for each ROI, (**g**) cluster radii and (**h**) number of localisations per cluster. Bars represent mean values and S.E.M. ns = not significant, *p ≤ 0.01, **p ≤ 0.001, ***p ≤ 0.0001, Mann-Whitney U Test.

Jurkat E6.1 T cells were transfected with the fluorescent photo-switchable fusion construct LAT-mEos3.2 36 h prior to imaging. *In vitro* immune synapses were formed by plating the transfected Jurkat T cells onto anti-CD3 and anti-CD28 coated glass coverslips containing embedded gold nanorods as fiducial markers. Cells were then fixed and washed before imaging by iPALM^10^. Non-overlapping regions of 2000 × 2000 × 600 nm were selected. Regions were selected randomly over the cell contact area. For the different conditions, cells were either allowed to settle onto uncoated coverslips (control), or allowed to form synapses for either 4 or 8 minutes before fixation. The data from these conditions is shown in Fig. 3. A total of n = 31 ROIs from at least n = 5 cells were analysed from each condition.

Figure 3a–c display representative analysed ROIs for the control condition (Fig. 3a), 4 minutes after activation (Fig. 3b) as well as 8 minutes after activation (Fig. 3c). See also projections of the region (SI Appendix Fig. S9). A first visual inspection of the resulting cluster maps shows that in all cases, LAT localisations and clusters exist in a 3D distribution; information that would be otherwise lost by 2D SMLM and cluster analysis. A substantial fraction of the LAT localisations lies further than 100 nm from the plasma membrane, which is the illumination depth in a typical TIRF-2D SMLM study. Therefore, the use of 3D super-resolution combined with 3D cluster analysis provides a more accurate and detailed description of the distribution of LAT at the T cell immunological synapse.

The total number of localisations per ROI significantly increases from control and early signalling (4 minutes time point) to a fully formed synapse (8 minutes time point) (Fig. 3d), consistent with LAT being recruited to the immune synapse following TCR triggering^33, 35^. We observe that LAT is clustered in resting cells, consistent with previous reports^32, 33^. Although the number of clusters remains similar between resting and activated cells (Fig. 3e), the other cluster descriptors significantly vary, suggesting the reorganisation of LAT at the nanoscale following activation. In particular, the percentage of localisations in clusters increases significantly between control (57.6 ± 17.7) and the 4 minute time point (69.3 ± 14.2, p = 0.0062), whereas there is no significant difference (p = 0.9349) between control and fully formed synapses at the 8 minute time point (Fig. 3f). This suggests a possible two stage process of LAT recruitment followed by subsequent dispersal. Figure 3g illustrates that cluster radii increase significantly in fully formed synapses compared to early signalling (from 25.6 ± 14.8 to 33.8 ± 19.5, p < 0.0001), as do the number of localisations per cluster (from 68.6 ± 143.0 to 75.3 ± 100.4, p < 0.0001) (Fig. 3h), again, consistent with previous reports of LAT recruitment and the subsequent generation of larger scale protein islands^32, 33, 36^.

**Figure 4:**
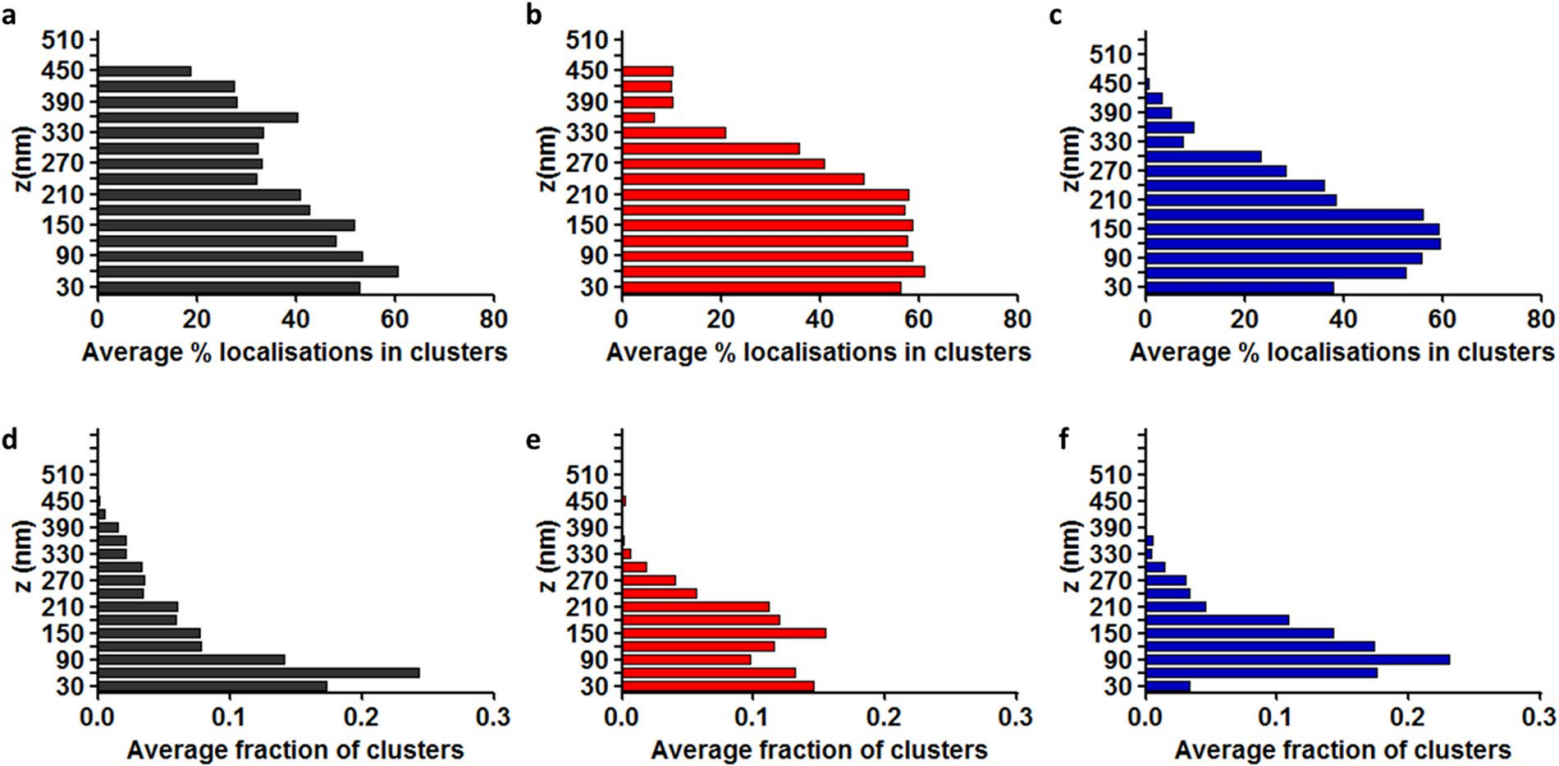
Histograms of the z distribution of LAT at the T cell immunological synapse. (**a**) Average percentage of localisations in clusters at each z plane for the control condition. (**b**) Average percentage of localisations in clusters at each z plane for 4 minutes post-activation. (**c**) Average percentage of localisations in clusters at each z plane for 8 minutes post-activation. (**d**) Average fraction of number of clusters at each z position for the control condition. (**e**) Average fraction of number of clusters at each z position for 4 minutes post-activation. (**f**) Average fraction of number of clusters at each z position for 8 minutes post-activation. Histogram bins are 30 nm.

The cluster analysis algorithm outputs the spatial position of all detected clusters. We are therefore able to quantify the clustering characteristics as a function of z for each condition. Figure 4a–c shows the variations in the percentage of localisations in clusters over z for activated cells in comparison with the control condition (bin width = 30 nm). Similarly, Fig. 4d–f summarize the average fraction of clusters located in layers of 30 nm in z from z = 0 nm to z = 600 nm for 4 minute and 8 minute time points compared to control conditions. Under control conditions, clusters are relatively evenly distributed in z, with a significant proportion located above 300 nm. Upon activation, clusters are recruited to the synapse interface, with the main population found around 150 nm above the coverslip at 4 minutes post-activation and around 90 nm above the coverslip by 8 minutes. As expected, there is a concurrent drop in the cluster population above 300 nm, consistent with the total number of clusters remaining approximately constant. This is indicative of a translocation of the higher population of clusters towards the plasma membrane over the course of activation.

## Discussion

3D SMLM is becoming an increasingly commonly used tool in biological sciences. Uniquely, this technique is capable of resolving the distribution of proteins on the nanoscale and with single molecule specificity. Unlike conventional microscopy, SMLM produces pointillist data sets which must be quantified using spatial point pattern analysis. However, most of the tools developed to date have been tailored for 2D SMLM. The cluster analysis tool presented here has been designed taking into account the specificities of 3D SMLM. One of the most important of these is to take account of the individual localisation precisions. Unlike in 2D, the localisation precisions assigned in 3D SMLM are not equal in each dimension due to the nature of the imaging process. The open source algorithm presented here takes as input 6 variables for each localisation: the x, y and z coordinates as well as their respective uncertainties. As output, it provides a full characterisation of the clustering, a visualisation of the cluster map and several key cluster descriptors, in particular, the clusters radii, percentage of localisations in clusters, number of clusters per ROI and number of localisations per cluster, as well as the variation of clustering over the z dimension. The computational cost of the presented method is relatively low, with one ROI in the Standard Condition analysed in about 20 minutes.

We have shown using simulated data the reliability of the method in a range of different clustering conditions. The method is appropriate provided that the data resembles the proposed Bayesian model, the principal point being that the clusters are approximately spherical and Gaussian in profile. Nonetheless, we have also demonstrated the validity of the algorithm in the context of other spherical shaped clusters (such as hard edges clusters). Overall, our results suggest that the model provides a robust analysis for a range of data sets. It enables reliable analysis over strong variations in the clustering parameters (such as unclustered localisations and cluster sizes) as well as non-Gaussian, hard edge clusters and non-circular ellipses. We also show that the analysis is relatively unaffected by a modification of the prior parameters. If spherical clusters are not reflective of the clustering behaviour, for instances for fibrous structures, other cluster analysis methods such as DBSCAN^37^ may be more suitable. However, these methods often require subjective user-supplied analysis parameters which may heavily bias the results, and which are not required by our Bayesian method. Also, as these alternative techniques do not rely on a model, they have been shown in 2D studies to be likely to be affected by an uneven background which is a recurring feature in 3D SMLM data sets. Alternatively, there are other methods which provide an overview, rather than a full description of the clustering, such as Ripley’s K-function^21, 22^ and Pair Correlation^19, 38^.

We have demonstrated the power of 3D cluster analysis using experimental data acquired by iPALM which uses both interferometric analysis and astigmatism to obtain quasi-isotropic localisation precision of approximately 10–30 nm in all three dimensions^10^. However, all 3D SMLM methods (e.g. Biplane, helical PSFs, astigmatism) produce data suitable for our cluster analysis. In this case, iPALM was used to investigate the 3D distribution of LAT at the T cell immunological synapse as a proof of concept.

**Figure 5:**
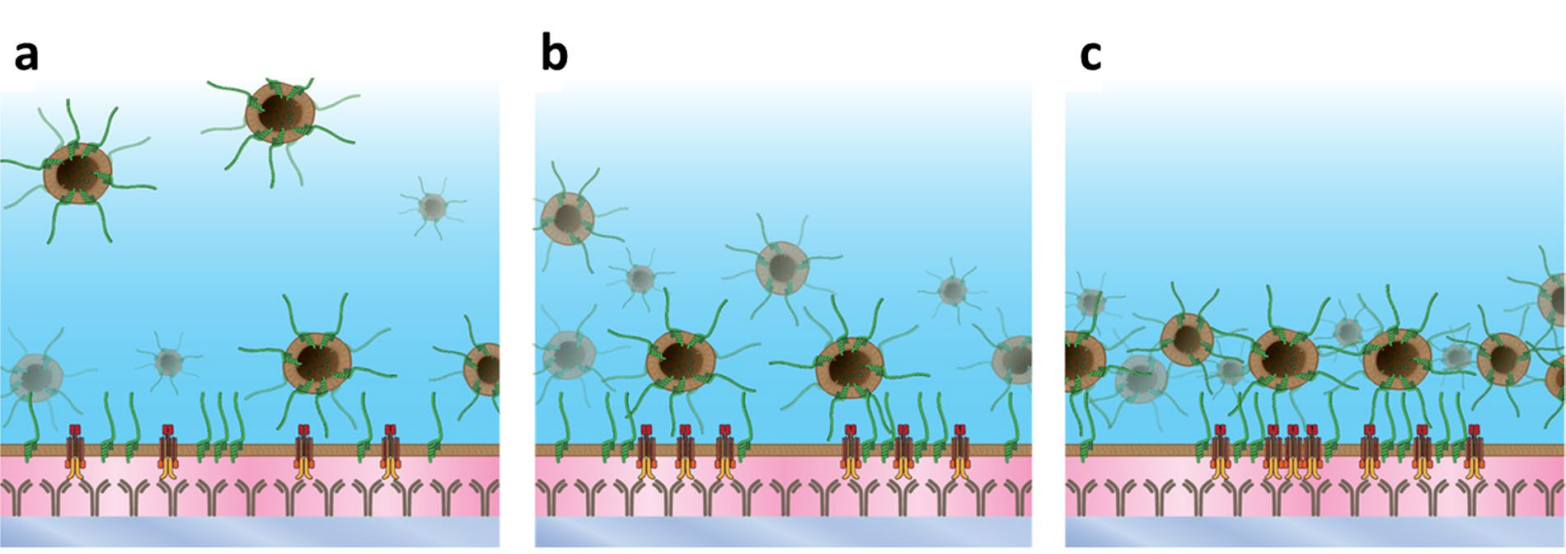
Model for LAT (green) recruitment following T cell activation through the TCR (yellow and red) pathway via antibodies (grey). (**a**) Control conditions showing LAT vesicles up to 500 nm in depth as well as pre-clustered LAT in the plasma membrane. (**b**) 4 minutes after activation showing the recruitment of LAT vesicles to the vicinity of the plasma membrane. (**c**) 8 minutes condition showing the accumulation of recruited vesicles proximal to the membrane forming a dense signalling platform.

LAT is a transmembrane adaptor protein required for T cell activation which exists in at least two distinct pools^35^. After stimulation through the TCR-CD3 complex, it is phosphorylated by the kinase ZAP-70 and subsequently acts as a recruitment platform for downstream signalling molecules such as SLP-76 and Vav. Our results confirm the presence of two distinct populations with a vesicular population existing up to 500 nm above the plasma membrane in resting cells. The second is a plasma membrane population which we observe to be pre-clustered in unstimulated cells, in agreement with previous reports by Williamson *et al*.^33^ and Lillemeier *et al*.^32^. Upon activation, our results suggest that the vesicular population is recruited to the immunological synapse interface, and within 4 minutes, the majority of LAT vesicles are located at approximately 150 nm above the membrane. By 8 minutes, the majority are located within 100 nm of the membrane. The large-scale recruitment of vesicles to close proximity of the plasma membrane, combined with the plasma membrane clusters themselves, creates an aggregated 3D signalling platform. This is evidenced by the observed increase in cluster radii, an increase in the number of localisations per cluster and the depopulation of clusters from deeper regions. We also observe an increase in the total number of localisations after activation, which could be the result of recruitment of vesicles between 4 and 8 minutes from beyond the imaging depth. A proposed model for the recruitment of LAT vesicles is shown in Fig. 5 and previous studies have shown that such a recruitment of LAT containing vesicles is dependent on the SNARE Vamp7^39^. While our data is consistent with the recruitment of LAT vesicles to the immune synapse from an intracellular pool, it does not discount the possibility of additional vesicles generated by membrane endocytosis. In fact, our data suggests the possible generation of LAT vesicles by endocytosis as evidenced by the small decrease in the number of LAT clusters at the plasma membrane post-activation.

In conclusion, we have developed a cluster analysis method for 3D SMLM data, and tested its applicability using simulated data as well as iPALM imaging of LAT organization in T cells. In the future, 3D SMLM will become more widely available and used routinely in the context of biological sciences as it provides a more accurate insight than conventional 2D SMLM which consists of a 2D projection of a 100 nm thick volume. While we have implemented a simple Bayesian model here, in principle, other more complex models are possible, for example, top-hat profile clusters or ellipses. Techniques such as these will allow SMLM to transition from providing visual illustration of the nanoscale architecture to providing reliable quantitative information and statistics for complex biological studies.

## Electronic supplementary material

Supplementary information
