# Supplementary material for: 3D Bayesian cluster analysis of super-resolution data reveals LAT recruitment to the T cell synapse
Source: Sci Rep. 2017 Jun 22;7:4077. doi: 10.1038/s41598-017-04450-w (PMC5481387; doi:10.1038/s41598-017-04450-w)
Supplement: Supplementary file 1 — Supplementary information [file 41598_2017_4450_MOESM1_ESM.pdf]

3D Bayesian cluster analysis of super-resolution data reveals LAT recruitment to the T cell synapse

Juliette Griffié[1], Leigh Shlomovich[2], David J Williamson[1], Michael Shannon[1], Jesse Aaron[3], Satya Khuon[3], Garth Burn[1], Lies Boelen[4], Ruby Peters[1], Andrew P Cope[5], Edward A. K. Cohen[2], Patrick Rubin-Delanchy[6] and Dylan M Owen[1]

[1]Department of Physics and Randall Division of Cell and Molecular Biophysics, King's College London
[2]Department of Mathematics, Imperial College London
[3] Advanced Imaging Center, Howard Hughes Medical Institute Janelia Research Campus, Ashburn, Virginia
[4]Department of Medicine, Imperial College London
[5]Department of Immunology, Infection and Inflammatory Disease, King's College London
[6]Department of Statistics, University of Oxford

Supplementary Information:

Analytical tool methodology

As explained in the main text, we assign to all localisations a density estimate, $L_{3D}(r)$, as an indication of the local clustering around each point. A localisation is defined as being a local maximum if it has been assigned the highest $L_{3D}(r)$ value in a sphere of the same radius r, centred on it. The choice of which specific maxima to consider as being associated with true cluster positions is based on their topographic prominence (TP) value rather than their absolute $L_{3D}(r)$ values, previously shown to improve performance[25]. We define the topographic prominence of a maximum in the context of pointillist data as the interval between its $L_{3D}(r)$ value and a base value (the key col value) which allows connectivity to higher maximum. The key col is defined as the lowest value of $L_{3D}(r)$ for which two maxima are connected. Two maxima are considered connected if a path exits between them using a step size smaller than the mean CSR nearest neighbour distance stepping only between points with L_{3D}(r) value above the key col value. We next apply a threshold T to the calculated TP values of each local clustering maximum. Only maxima with a TP value above T are seen as identifying clusters, hence by varying the TP threshold, different cluster proposals are generated. Finally, once a maximum above the threshold has been identified, the attribution of localisations to the associated cluster relies only on connectivity to the selected maximum.

Cell culture and transfection:

Jurkat T E6.1 cells were cultured in RPMI supplemented with Glutamax, 10% Foetal Calf Serum (FCS), 100 mg/ml Penicillin and 100 μg/ml streptomycin, incubated at 37˚C in a 5% CO_2 incubator. Cells were electroporated with Amaxa Nucleofector Kit V (Lonza, Germany). Plasmid DNA contained LAT fused to mEos3.2 under the control of the CMV promoter. Once transfected, the cells were incubated in medium for 36 hours at 37˚C to allow expression of tagged LAT.

Synapse formation and fixation:

The coverslip was coated with anti-CD3 (2 μL/mL) and anti-CD28 (5 μL/mL) in Hank's Balanced Salt Solution (HBSS) before being incubated for one hour. The coverslip was then washed three times with HBSS before being used for synapse formation.

The transfected Jurkat T cells were washed in HBSS and spun down twice before being suspended at a concentration of 1×10^6 cells per mL for imaging. The cells were allowed to settle on the coverslip (t=0 s for the activation time course). The coverslip was placed back in the incubator for 4 or 8 minutes depending on the condition. For the control case, the same steps as the 8 minute case were followed, with uncoated coverslips. Cells were then fixed at room temperature using a 2-step protocol. First, cells were fixed using 3% paraformaldehyde (PFA) in 80 mM kPIPES at a pH of 6.8 for 5 minutes then washed twice with HBSS. The coverslip was then placed in 3% PFA in 100 mM NaB_4O_2 at a pH of 11 for 10 minutes and washed three times with HBSS before imaging. Finally, samples were mounted between a lower 25 mm diameter #1.5 coverslip (also containing the fiducial markers) and an upper 18 mm diameter #1.5 coverslip[10]. Coverslips were bonded together using epoxy (3 M) and sealed to prevent buffer evaporation using Vaseline (Unilever).

Imaging:

The imaging was performed on an iPALM microscope at the Advanced Imaging Center at the Howard Hughes Medical Institute's Janelia Research Campus [10]. 30,000 frames were acquired, using an integration time of 50 ms each. Two lasers were used during the acquisition: a pulsed 405 nm laser, delivering 5-30 W/cm^2 to photoconvert the fluorescent protein, as well a continuous wave (CW), 561 nm laser at 1 kW/cm^2 for fluorescence readout. The 405 nm laser pulse width was continuously increased during acquisition to maintain an approximately constant number of visible molecules in each frame. Fluorescence was collected in the range 570 – 615 nm (FF01-593/40-25, Semrock).

Image processing:

The raw data from the acquisition was processed by the Peak Selector software package[5]. Lists of localisations were extracted from the raw data, which was, in turn, filtered by uncertainty (to less than 30 nm in all dimensions). Drift correction in x,y and z and tilt correction was performed using fiducials. Finally, the data was cropped into 2 x 2 x 0.6 μm ROIs.

Statistics:

P values were calculated using an unpaired, two-tailed, Mann-Whitney U-test in Prism software.

Code availability:

All code related to this analysis is be available at: GitHub public “OwenlabKCL” page.

Supplementary Figures

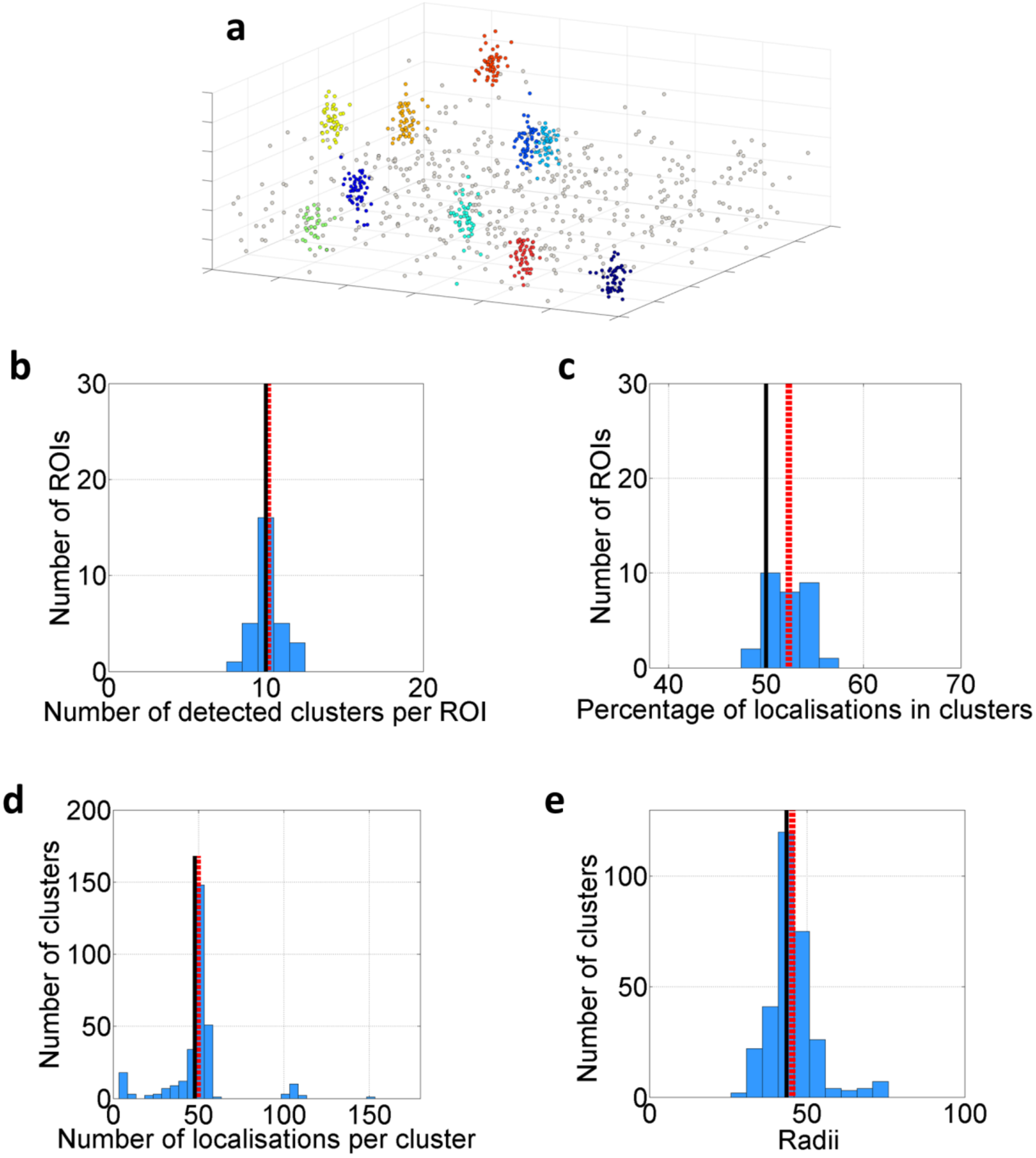

SI appendix Figure 1: *3D Bayesian cluster analysis of n = 30 simulated data sets in the uneven background condition (non-clustered localisations fitted to a (5:2) β distribution) (a) Representative 3D cluster map with the simulated value in each case shown as a black line and mean detected number as the red dashed line. (b) Number of detected clusters per ROI, (c) Percentage of localisations detected in clusters, (d) Number of detected localisations per cluster and (e) cluster radii.*

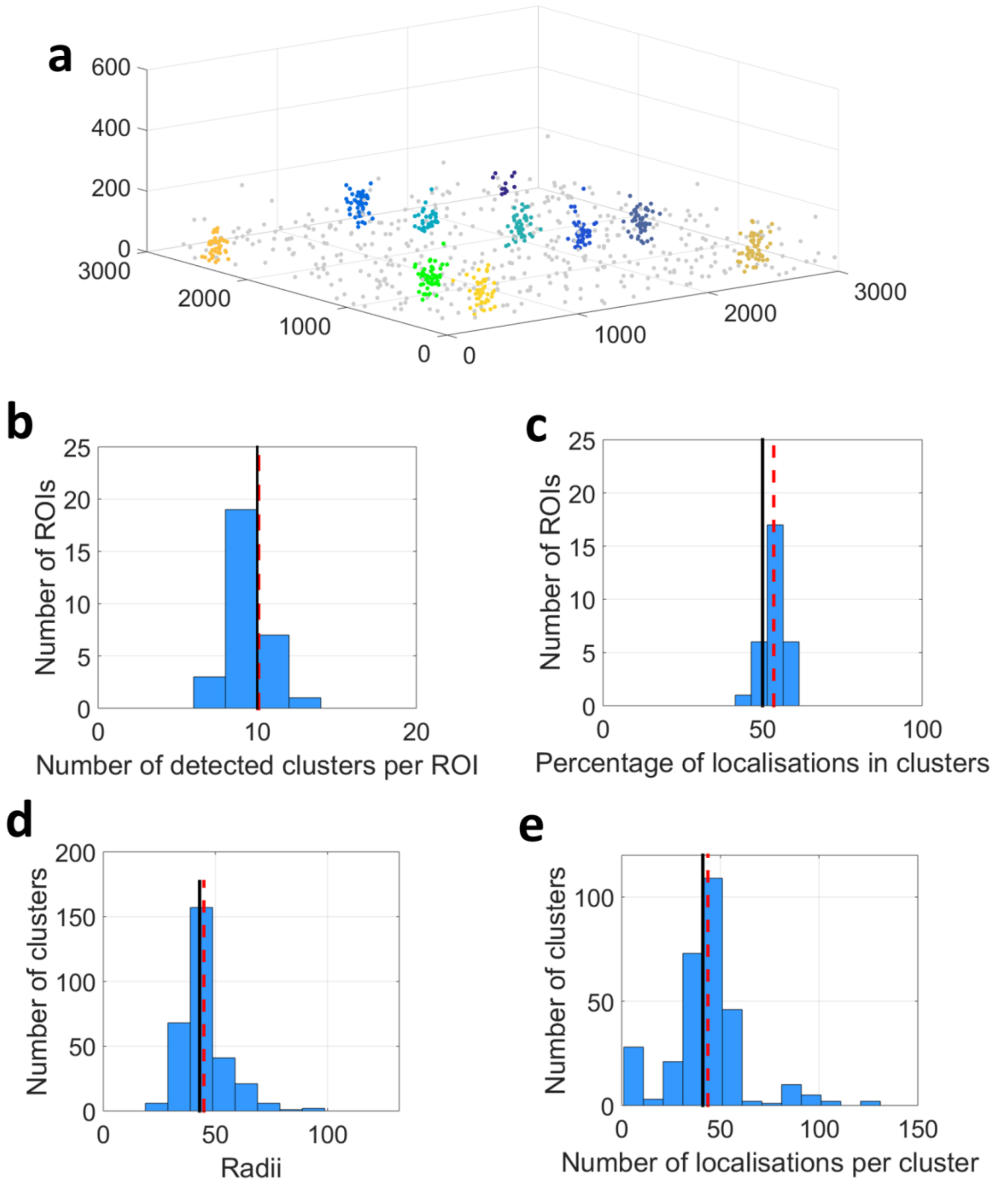

SI appendix Figure 2: *3D Bayesian cluster analysis of n = 30 simulated data sets of a distribution proximal to the coverslip with Beta distribution used as a prior for the model (a) Representative 3D cluster map with the simulated value in each case shown as a black line and mean detected number as the red dashed line. (b) Number of detected clusters per ROI, (c) Percentage of localisations detected in clusters, (d) cluster radii (nm), e) Number of detected localisations per cluster.*

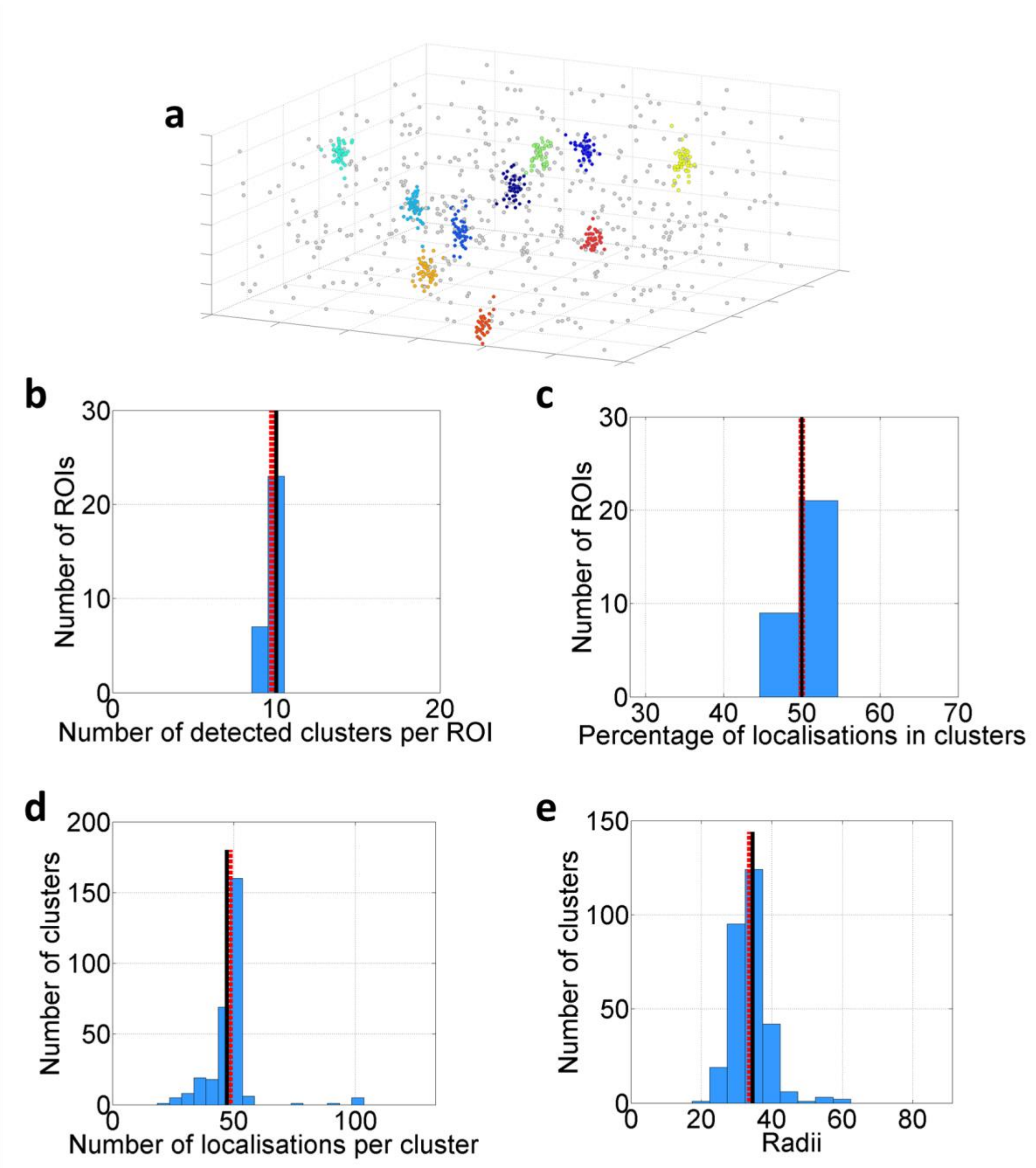

SI appendix Figure 3: *3D Bayesian cluster analysis of n = 30 simulated data sets of the standard condition with hard edge clusters, (a) Representative 3D cluster map with the simulated value in each case shown as a black line and mean detected number as the red dashed line. (b) Number of detected clusters per ROI, (c) Percentage of localisations detected in clusters, (d) Number of detected localisations per cluster and (e) cluster radii (nm).*

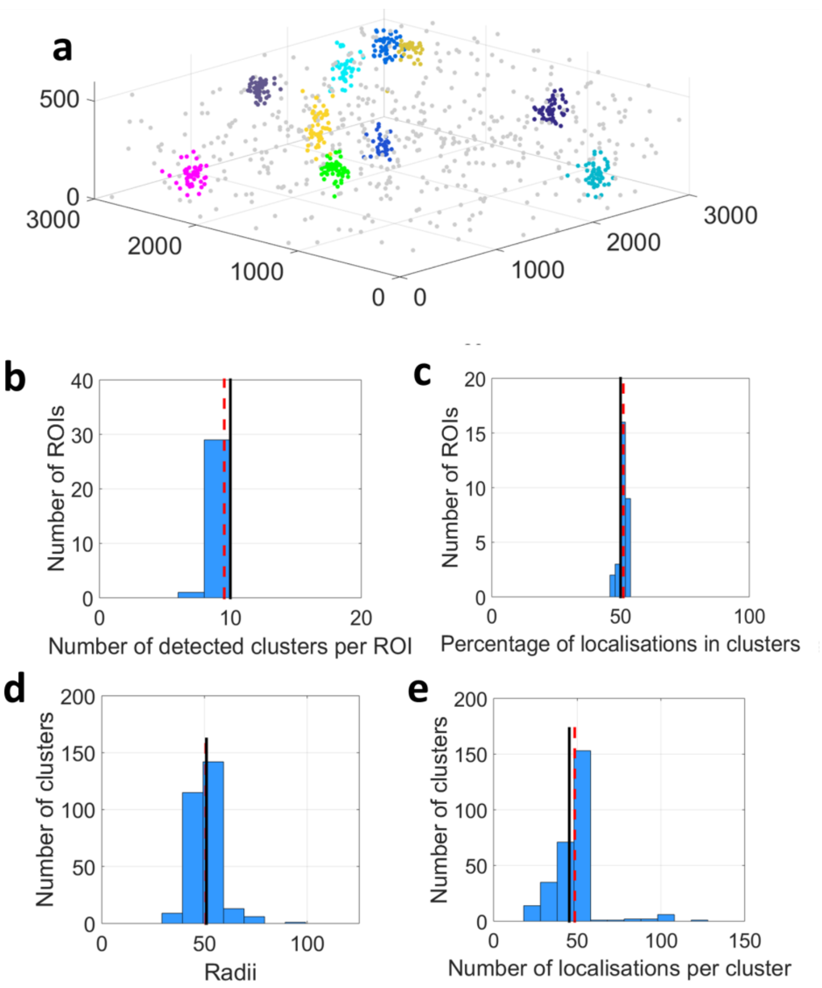

SI appendix Figure 4: *3D Bayesian cluster analysis of n = 30 simulated data sets in the case of elliptic clusters (randomly oriented towards the x, y or z axis) with a ratio (1,1,2) for an ellipse along the z axis for instance (a) Representative 3D cluster map with the simulated value in each case shown as a black line and mean detected number as the red dashed line. (b) Number of detected clusters per ROI, (c) Percentage of localisations detected in clusters, (d) Number of detected localisations per cluster and (e) cluster radii (nm).*

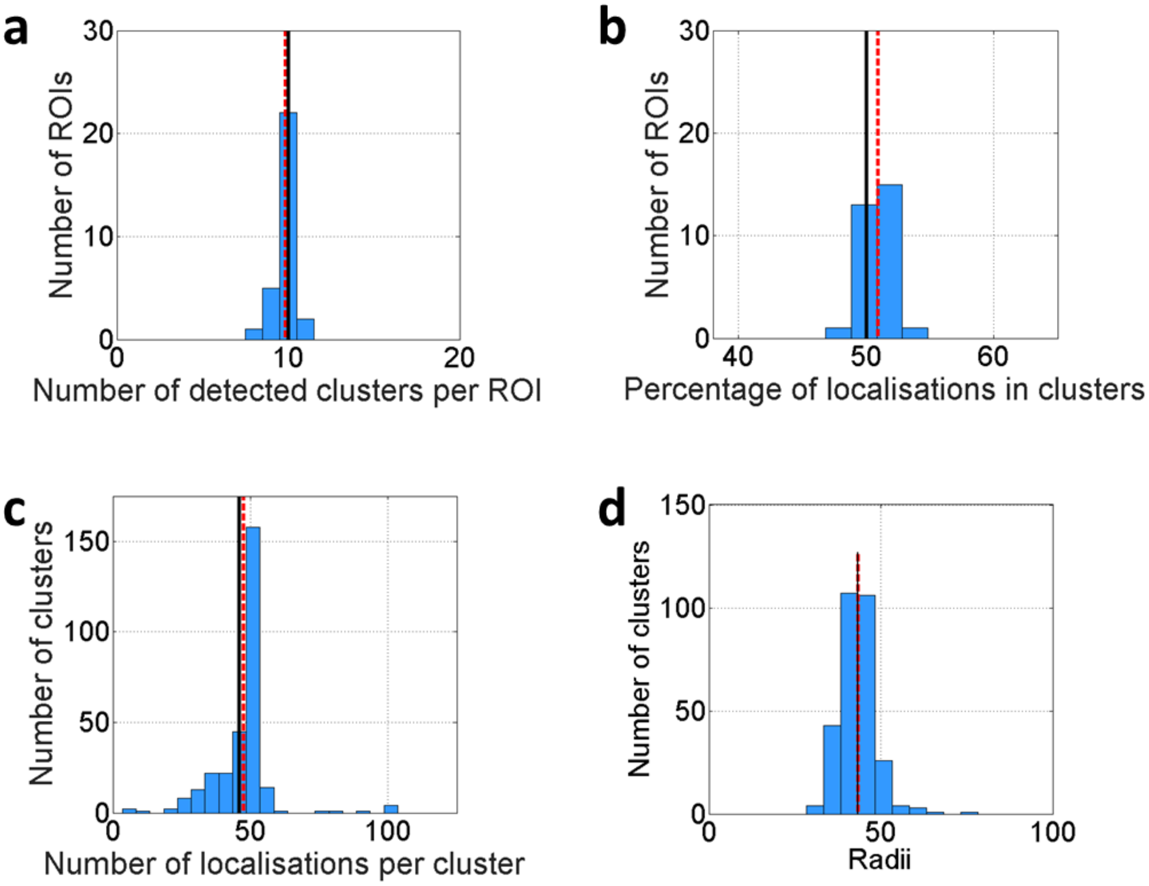

SI appendix Figure 5: *Standard condition with the model un-clustered localisation ratio set at 25%. The simulated value in each case is shown as a black line and the mean detected number as the red dashed line. (a) Number of detected clusters per ROI, (b) Percentage of localisations detected in clusters, (c) Number of detected localisations per cluster and (d) cluster radii (nm).*

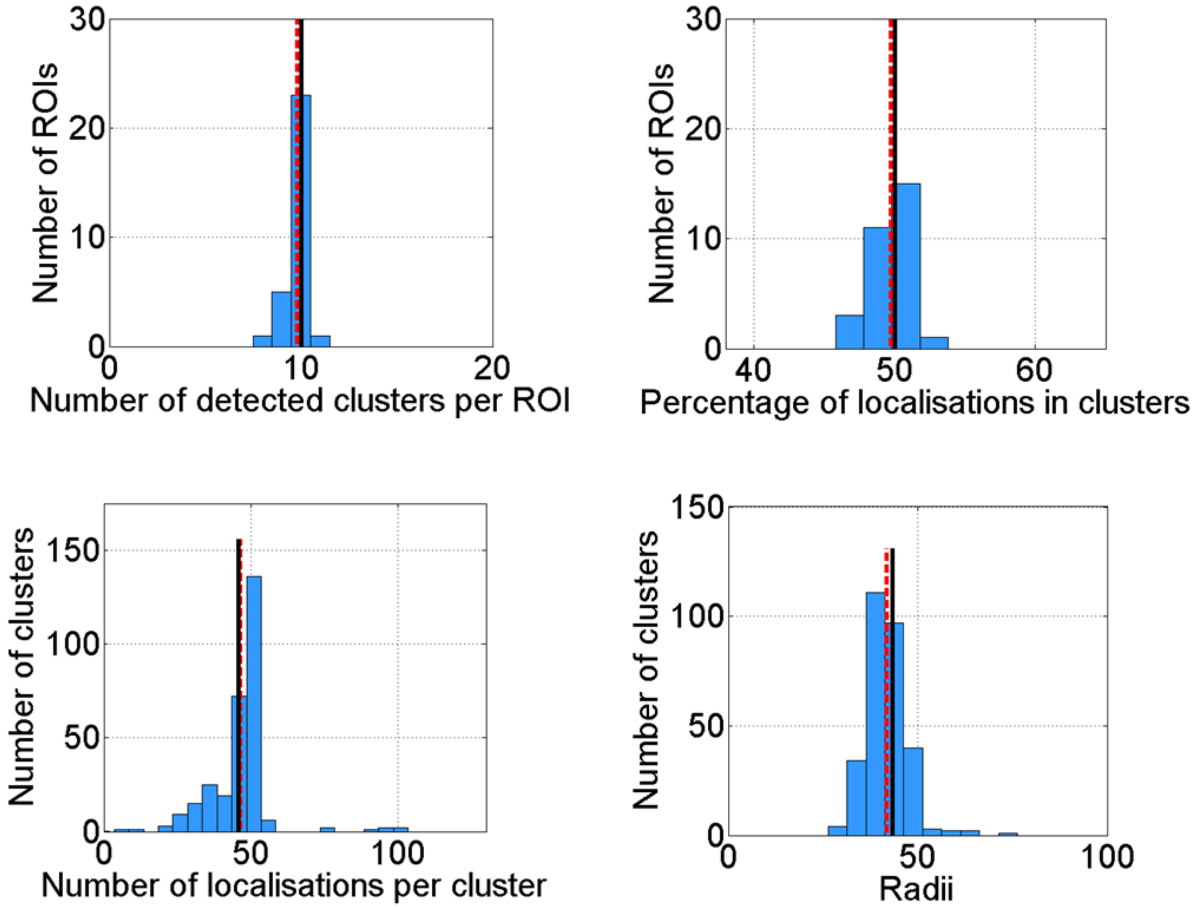

SI appendix Figure 6: *Standard condition with the model un-clustered localisation ratio set at 75%. The simulated value in each case is shown as a black line and the mean detected number as the red dashed line. (a) Number of detected clusters per ROI, (b) Percentage of localisations detected in clusters, (c) Number of detected localisations per cluster and (d) cluster radii (nm).*

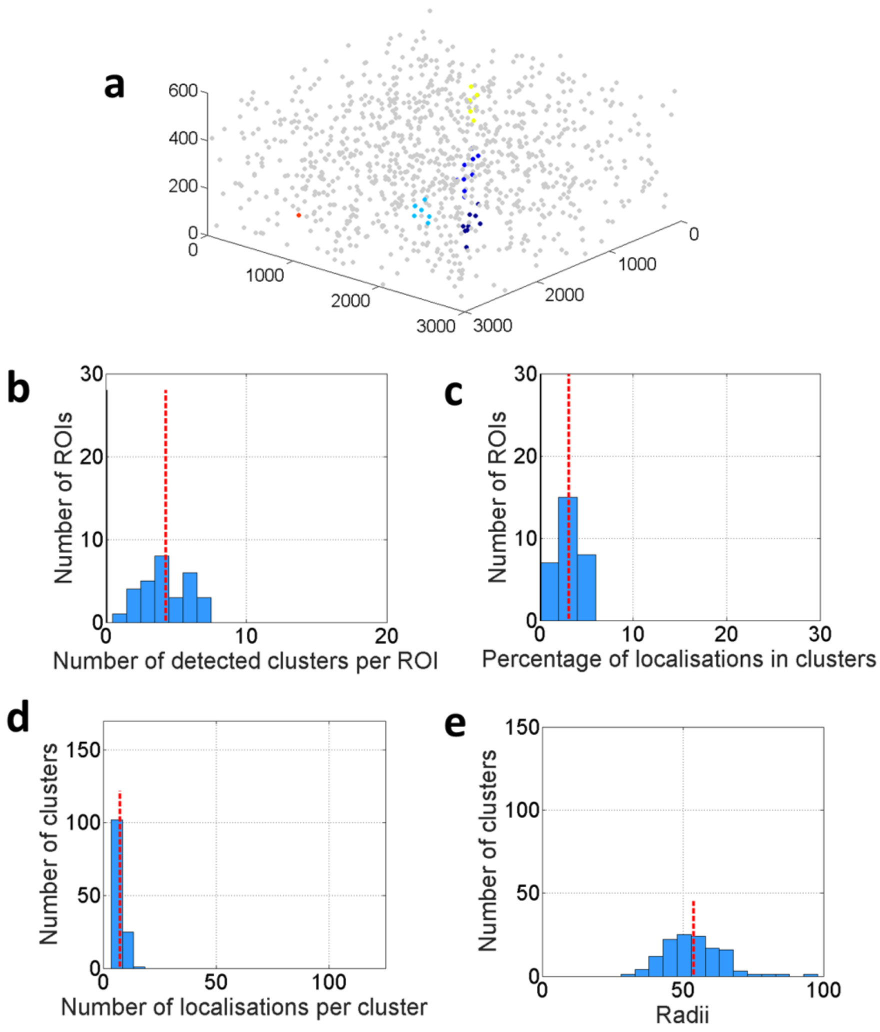

SI appendix Figure 7: *3D Bayesian cluster analysis of n = 30 simulated data sets of CSR distribution with 1000 localisations (a) Representative 3D cluster map with mean detected number as the red dashed line. (b) Number of detected clusters per ROI, (c) Percentage of localisations detected in clusters, (d) Number of detected localisations per cluster and (e) cluster radii (nm).*

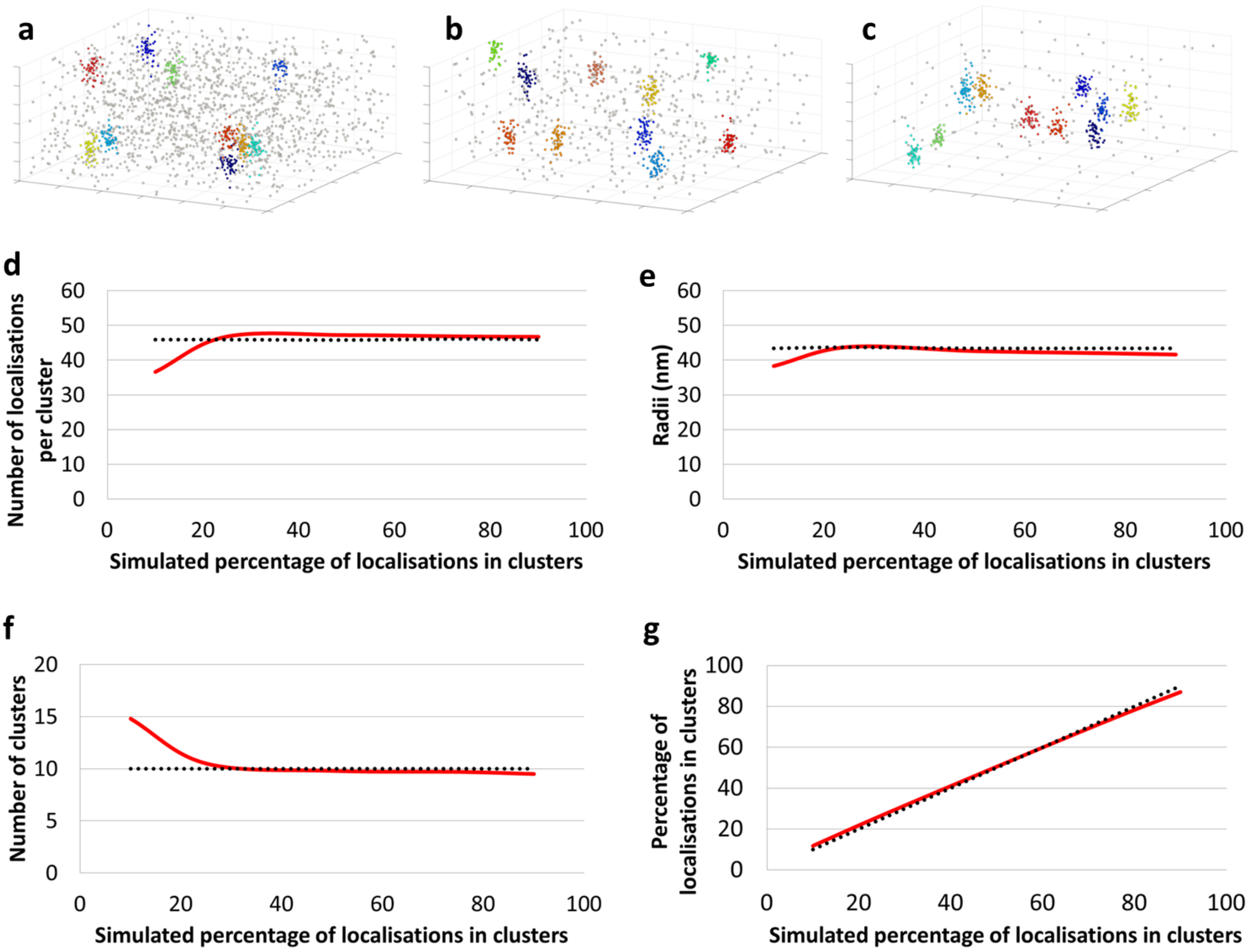

SI appendix Figure 8: *Bayesian cluster analysis on simulated data sets under the Standard Conditions while varying the percentage of localisations in clusters from 10% to 90%. (a) Representative cluster maps (from n = 30) with 25% localisations in clusters within the 3000 x 3000 x 600 nm ROI. (b) Same condition but with 50% localisations in clusters within the ROI. (c) Same condition but with 75% localisations in clusters within the ROI. (d) Number of detected localisations per cluster as a function of the percentage of localisations in clusters, (e) cluster radii as a function of the percentage of localisations in clusters, (f) number of detected clusters per ROI as a function of the percentage of localisations in clusters and (g) percentage of localisations detected in clusters as a function of the percentage of localisations in clusters. Red = results of the analysis, black = simulated values.*

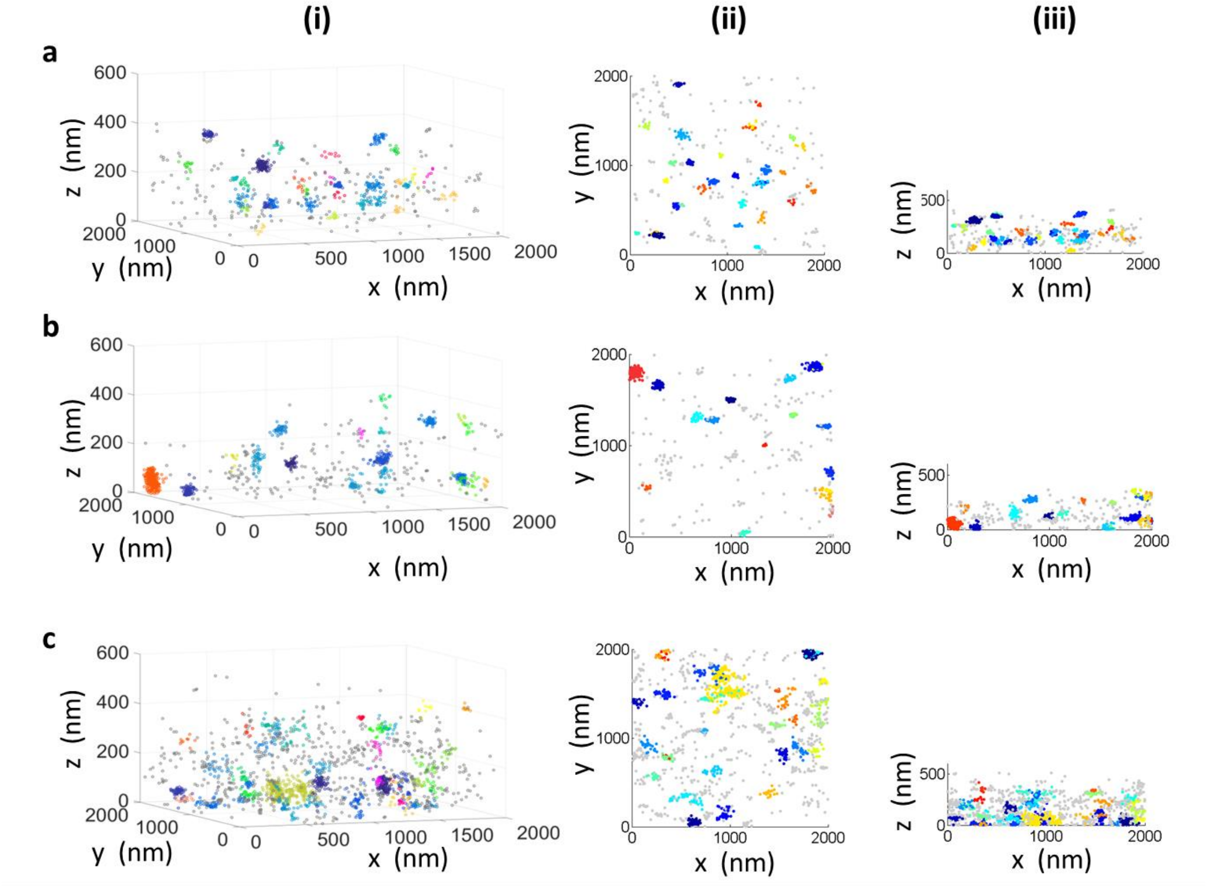

SI appendix Figure 9: *Representative cluster maps from a) control condition, b) 4 minutes and c) 8 minute synapses showing i) 3D representation of the clusters, ii) projection of the data in x-y and iii) projection of the data in x-z.*
